# Interventional treatment of peripancreatic aneurysms: can one strategy fit all?

**DOI:** 10.1186/s42155-025-00533-2

**Published:** 2025-03-19

**Authors:** Marilia B Voigt, Patrick A Kupczyk, Alexander Kania, Carsten Meyer, Julia Wagenpfeil, Tatjana Dell, Claus-Christian Pieper, Julian A Luetkens, Daniel Kuetting

**Affiliations:** 1 Department of Diagnostic and Interventional Radiology, University Hospital Bonn, Venusberg-Campus 1, Bonn, Germany; 2 Department of Visceral and Vascular Surgery, University Hospital Bonn, Bonn, Germany

**Keywords:** Visceral artery aneurysms, Peripancreatic arteries, Visceral artery stenosis, Aneurysm-to-vessel ratio

## Abstract

**Purpose:**

To identify the frequency and association of visceral arterial (VA) stenosis in peripancreatic aneurysms (PPAs) and to develop a uniform, more detailed treatment strategy for PPAs in case of accompanying VA stenosis, as current guidelines do not adequately address this constellation.

**Materials and methods:**

Patients with PPAs diagnosed at a tertiary care hospital were retrospectively analyzed. In case of multiple PPAs, the aneurysm with the highest aneurysm-to-vessel ratio (AVR) within the celiac-mesenteric collateral circulation was classified as the primary aneurysm and categorized as "critical" or "non-critical" based on the risk of organ ischemia.

Celiac artery and superior mesenteric artery stenoses were graded as low (< 50%), high (> 50%), or total occlusion. Treatment strategies were based on VA stenosis severity, aneurysm classification, and morphology. Treatment strategies included endovascular, surgical and watch-and-wait management.

**Results:**

Thirty-one patients with PPAs were included with a total of 53 aneurysms; mean aneurysm size: 12.5 ± 7.9 mm (range 5–38 mm), AVR: 3.5 ± 2.1 (range 1–11.3). The superior and inferior pancreaticoduodenal arteries as well as the pancreaticoduodenal arcade were affected in most cases (67.9%). AVR was significantly higher in cases of aneurysm rupture (6.2 ± 2.8; *p* = 0.031). Celiac artery stenosis was present in 87.1%.

Aneurysm size and occurrence of active bleeding did not correlate (*p* = 0.925). 11 patients presented with critical aneurysms, with 10 patients requiring individually tailored treatment. Non-critical aneurysms were treated with coil embolization in most cases.

**Conclusion:**

CA stenosis, aneurysm position, and AVR significantly influence treatment decisions. Individualized approaches based on anatomical and hemodynamic factors are needed in PPA treatment.

## Introduction

Visceral artery (VA) aneurysms are a comparatively rare disease entity with an incidence of approximately 0.01—0.2%. While all visceral branches can be affected, VA aneurysms are most frequently found in the splenic and hepatic artery [1]. One of the rarest subgroups are gastroduodenal and pancreaticoduodenal aneurysms, which can be summarized as peripancreatic aneurysms (PPAs) – accounting for 2–10% of VA aneurysms [2–4]. PPAs are reported to have a high rupture rate of up to 70%, leading to life-threatening complications [5]. In an emergency setting, mortality rates for actively bleeding PPAs are high (up to 75%) [5–8]. Consecutively, an accepted clinical practice is exclusion of PPA upon diagnosis regardless of the size or aneurysm type [9].

The association between visceral artery (VA) stenosis and PPAs remains a topic of ongoing investigation. Current literature is limited, primarily consisting of small case series [10, 11].

The prevailing view suggests that aneurysms in the peripancreatic region are predominantly caused by trauma, inflammatory processes, or infections [10, 12–14]. However, the hypothesis of hyperdynamic collateral circulation proposes a potential link between the celiac artery (CA) and superior mesenteric artery (SMA) stenosis and an increased risk of PPA development [15–17]. Additionally, increased blood flow in the PDA, as a result of VA stenosis causes increased vessel wall shear stress [17].

Atherosclerosis and vessel compression account for the majority of CA stenoses [18]. The latter are caused mainly by the median arcuate ligament, rarely by tumors [19].

While current literature almost unanimously recommends the elimination of PPAs regardless of size, there is little information on how to manage concomitant VA stenoses. Thus, there is an unmet need for more refined therapy concepts.

The aim of this study was to identify the frequency and association of VA stenoses in patients with PPAs and to develop a uniform, more differentiated recommendation algorithm for the treatment of VA stenoses in these patients.

## Materials and methods

This retrospective observational study was conducted with the approval of the local ethics committee under application number 303/16, which granted a waiver of informed consent.

### Patient selection

A retrospective analysis was conducted utilizing the internal hospital databases. Radiological reports from January 2014 to December 2023 were searched using a predefined keyword list encompassing terms related to peripancreatic location ("pancreaticoduodenal aneurysm”, “gastroduodenal aneurysm"), potential collateral pathways ("Bühler anastomosis," "Rio Branco arcade"), and the involved vessels ("celiac axis," "celiac artery," "superior mesenteric artery").

Patient inclusion criteria included availability of an abdominal CT scan with an arterial contrast phase and diagnosis of a PPA based on the radiological reports. Exclusion criteria were prior surgical or interventional treatment, prior radiation therapy affecting the peripancreatic region and/or underlying connective tissue diseases associated with the development of arterial aneurysms.

### Patient demographics

A total of 31 patients (including 12 women and 19 men) were diagnosed with at least one visceral aneurysm in the peripancreatic region: superior PDA, inferior PDA, pancreaticoduodenal arcade or GDA. Mean patient age was 65 years (range 36 to 84 years) at the time of diagnosis.

### Data collection and definition

Patient demographics, comorbidities, medical history, and aneurysm characteristics, including size, morphology, presence of plaques, and rupture, were documented. Aneurysms located within the celiac-mesenteric collateral circulation (gastroduodenal artery (GDA), superior pancreaticoduodenal artery (PDA), inferior PDA, pancreaticoduodenal arcade, CA and SMA), were classified as primary aneurysms. In cases where multiple aneurysms were present in these vessels, only the aneurysm with the highest aneurysm-to-vessel ratio (AVR) was designated as the primary aneurysm. Additional smaller aneurysms or those located outside the celiac-mesenteric collateral circulation were categorized as secondary aneurysms.

For each patient, one primary aneurysm was defined and classified into two categories based on its position: "critical" or "non-critical." A critical aneurysm was defined as one where embolization of the carrier vessel was supposed to result in downstream organ ischemia due to insufficient collateral circulation, respectively if the aneurysm was positioned in the only vessel connecting two flow areas. Non-critical aneurysms were defined to have adequate collateral circulation, mitigating the risk of ischemia even if the carrier vessel was occluded. It was also defined for secondary aneurysms whether they were “critical” or “non-critical”.

Aneurysms were defined as localized arterial enlargements exceeding 50% of the native vessel diameter, confirmed through both double-angled and cross-sectional measurements. True aneurysms were defined as vessel enlargements, where the vascular wall was involved, whereas the enlargement did not involve the vascular wall in pseudoaneurysms. The AVR was calculated for all primary and secondary aneurysms. Primary aneurysms in critical positions were further stratified into two groups based on AVR: those with an AVR < 3 and those with an AVR > 3.

The presence of VA stenoses was assessed in the CA and the SMA. Stenotic lesions were categorized into three groups: low-grade stenoses (< 50%), higher-grade stenoses (> 50%), and total occlusions. Underlying etiologic pathogenesis of the stenoses was documented.

### Treatment

The treatment administered to each patient was documented. All procedures were performed by the same multidisciplinary team of interventional radiologists (3) and vascular surgeons (2). At our tertiary referral center, around 1500 fluoroscopy-guided interventions and 1000 vascular surgeries are performed annually.

Therapy for the primary aneurysms was individualized based on the presence and severity of VA stenosis as well as the supposed etiology of stenosis, the aneurysm’s position (critical versus non-critical), configuration, type, size, morphology, and the presence of active bleeding.

Treatment options included:

1. Revascularization of VA stenosis: Achieved through endovascular stenting or surgical techniques such as aorto-truncal bypass or external surgical division of the median arcuate ligament (MAL).
2. Aneurysm exclusion with preservation of the carrier vessel: Performed via endovascular placement of stent grafts, stent/balloon remodeling, or surgically with aneurysm exclusion coupled with vascular reconstruction. Stent grafting is an endovascular technique used to exclude aneurysms from circulation by deploying a covered stent across the aneurysm neck or along the affected vessel segment. Stent grafts consist of a metallic scaffold covered with a biocompatible fabric, effectively sealing the aneurysm and redirecting blood flow through the graft lumen. Compared to coil embolization, stent grafting is particularly advantageous in fusiform or dissecting aneurysms and in cases where maintaining perfusion of side branches is not critical. However, long-term durability depends on adequate graft apposition, endothelial integration, and the absence of endoleaks. Balloon remodeling was used to enhance coil embolization in aneurysms, particularly in cases with a wide neck, or in cases with contraindications to anticoagulation. A temporary compliant balloon is inflated within the parent artery adjacent to the aneurysm neck during coil deployment. This temporarily alters blood flow, stabilizes the coils within the sac, and prevents coil prolapse into the parent vessel. Once adequate packing is achieved, the balloon is deflated and removed, restoring normal vessel patency while maintaining aneurysm occlusion. In stent-assisted remodeling, a permanent self-expanding stent was deployed across the aneurysm neck before coil placement. The stent serves as a scaffold, preventing coil herniation and promoting endothelialization, which can lead to progressive aneurysm exclusion over time.
3. Aneurysm exclusion without preservation of the carrier vessel: Primarily executed through endovascular coil embolization in front-and-back-door technique or surgically through aneurysm ligation without subsequent vascular reconstruction. Coil embolization involves embolizing both the inflow ("front door") and outflow ("back door") vessels to completely exclude an aneurysm from circulation. Front-door embolization blocks the primary feeding artery, reducing aneurysm pressure, while back-door embolization prevents retrograde filling via collateral pathways. Coils, liquid embolics, or vascular plugs are used for durable occlusion. Indications usually were saccular aneurysms with a well-defined neck, allowing stable coil placement, and an intact coagulation system.
4. CA Stenting involved the placement of a self-expanding stent to restore blood flow.

Unlike balloon remodeling, stent-assisted techniques required dual antiplatelet therapy (DAPT) to minimize the risk of in-stent thrombosis: We administered a minimal of 4 weeks of clopidogrel and lifelong aspirin for maintenance therapy.

### Statistical analysis

Frequencies and percentages were used to represent discrete variables. Data are shown as mean. Significance level was set at α = 0.05, determined by unpaired t-test.

## Results

### Clinical features

The majority of patients were diagnosed with one aneurysm in the celiac-mesenteric circulation (*n* = 19, 61.3%), six patients had two PPAs (22.6%). Less commonly, three (2), four (1) or even five (2) PPAs were diagnosed (6.5%, 3.2% and 6.5%, respectively). Hypertension (*n* = 19, 61.3%) and dyslipidemia (*n* = 16, 51.6%) were present frequently within the study population. In one patient, there was diagnosed a peptic ulcer of the stomach. Further patient characteristics are listed in Table 1.

**Table 1:** Patient characteristics

Patient characteristics (*n* = 31)	
Mean age in years	65 (36—84)
Gender	
Female	12 (38.7%)
Male	19 (61.3%)
Smoking	10 (32.3%)
Overweight	14 (45.2%)
Hypertension	19 (61.3%)
Dyslipidemia	16 (51.6%)
Peripheral artery disease	9 (29.0%)
Abdominal aortic aneurysm	6 (19.4%)
Type II diabetes	6 (19.4%)
Peptic Ulcers	1 (3.2%)

### VA Steno-occlusions

We identified 31 stenotic or occlusive lesions in the celiac-mesenteric circulation in total. They involved the CA in 27 cases (87.1%), and the SMA in 4 cases (12.9%). There was a notably high incidence of CA stenoses > 50% and occlusions, occurring in 26 patients (83.9%). In contrast, all detected SMA stenoses were < 50%. 23 patients (74.2%) had a CA and SMA stenosis and 4 patients had only CA stenosis (12.9%). Stenoses in other abdominal arteries were exceedingly rare, with only one patient exhibiting a subtotal atherosclerotic stenosis of the hepatic artery. In four patients, no stenosis was detected.

The most common underlying cause of CA stenosis was compression by the MAL, observed in 16 cases (59.3%), followed by atherosclerotic plaques in 9 cases (33.3%). Tumorous compression and inflammatory changes were each identified as causal in one patient (3.7%). All SMA stenoses were attributed to atherosclerosis.

### Aneurysm characteristics

In total, 53 aneurysms of the celiac-mesenteric circulation were detected in 31 patients. The mean aneurysm size was 12.5 mm (range: 5–38 mm ± 7.9 mm) with a mean AVR of 3.5 ± 2.1 (range 1–11.3). Aneurysm configuration was saccular in 60.4% (*n* = 32) and fusiform in 39.2% (*n* = 21). Presence of calcification and thrombi within the aneurysms was low with 13.2% and 15.1%, respectively. Of the identified aneurysms, 47 (88.7%) were true aneurysms and 6 (11.3%) pseudoaneurysms. The aneurysms were located in the superior/inferior PDA or pancreaticoduodenal arcade in 36 cases (67.9%), in the GDA in 8 cases (15.1%), in the CA in 6 cases (11.3%) and in the SMA in 3 cases (5.7%).

In the primary aneurysms, mean AVR was 4.04 ± 2.4 (range 1.75—11.33). AVR was significantly higher in cases with an aneurysm rupture (6.2 ± 2.8; *p* = 0.031). Aneurysm size and occurrence of active bleeding did not correlate (*p* = 0.925). In 11 patients (35.4%), primary aneurysms were located in critical positions. The primary aneurysms affected the superior PDA in 12 cases (38.7%), inferior PDA in 10 cases (32.3%), the pancreaticoduodenal arcade in 3 cases (9.7%) and the GDA in 6 cases (19.4%). Characteristics of primary aneurysms are documented in Table 2.

All secondary aneurysms were true aneurysms with an AVR < 3. There were no secondary aneurysms with a critical position.

**Table 2:** Primary aneurysm characteristics

Case	Sex f = female m = male	Comorbidities	Location primary aneurysms	AVR	Diameter (mm)	Aneurysm type	Higher grade CA stenosis	Active bleeding	Critical aneurysm localization	Treatment	Number of additional secondary aneurysms
1	m	Hypertension Dyslipidemia	sPPA	3	12	true	yes	no	yes	Balloon /coil remodeling of aneurysm	-
2	m	Smoking PAD	sPDA	2.3	9	true	yes	no	yes	Stenting CA	-
3	f	Overweight Hypertension Dyslipidemia PAD	sPDA	2.7	8	true	yes	no	yes	Stenting CA	-
4	f	Dyslipidemia	iPDA	6,5	13	true	yes	no	no	Coil embolization aneurysm + carrier vessel	-
5	f	Smoking Hypertenion Dyslipidemia PAD	sPDA	5	5	pseudo	yes	yes	no	Coil embolization aneurysm + carrier vessel	-
6	f	Hypertension PAD Peptic Ulcer	sPDA	4	8	pseudo	yes	yes	no	Coil embolization aneurysm + carrier vessel	-
7	m	Dyslipidemia	iPDA	2.2	11	true	yes	no	yes	Stenting CA + MAL section	1
8	m	-	iPDA	2.6	13	true	yes	no	no	Coil embolization aneurysm + carrier vessel	4
9	m	-	GDA	2.6	13	true	no	no	no	Coil embolization aneurysm + carrier vessel	1
10	m	Smoking	GDA	2.2	13	true	yes	no	no	Coil embolization aneurysm + carrier vessel	-
11	m	Hypertension Dyslipidemia	GDA	2.5	10	true	no	no	no	Coil embolization aneurysm + carrier vessel	1
12	m	-	arcPDA	5	10	pseudo	yes	yes	no	Coil embolization aneurysm + carrier vessel	-
13	m	Smoking Overweight Hypertension Dyslipidemia PAD Diabetes	arcPDA	11,3	34	true	no	no	no	Coil embolization aneurysm + carrier vessel	-
14	m	Hypertension Dyslipidemia	sPDA	3,5	7	true	no	no	no	Coil embolization aneurysm + carrier vessel	-
15	m	Hypertension	sPDA	11	11	true	yes	yes	yes	Open ligature of aneurysms + MAL section + aorto-truncal bypass	4
16	m	Smoking Overweight Hypertension Dyslipidemia PAD Diabetes	iPDA	2	6	true	yes	no	no	Coil embolization aneurysm + carrier vessel	-
17	m	Smoking	GDA	5,8	35	true	yes	yes	yes	Exclusion of aneurysm with stent graft	1
18	f	Hypertension Dyslipidemia	iPDA	2,8	9	true	yes	no	no	Coil embolization aneurysm + carrier vessel	-
19	f	Overweight	sPDA	1,8	7	true	yes	no	no	Coil embolization aneurysm + carrier vessel	-
20	f	Overweight	arcPDA	5,5	11	true	yes	no	yes	Stent / coil remodeling of aneurysm	1
21	m	Smoking Overweight Hypertension Dyslipidemia PAD	sPDA	2	6	true	yes	no	yes	Stenting CA	2
22	m	-	sPDA	6,5	13	pseudo	yes	no	no	-	-
23	m	Overweight Hypertension Diabetes	GDA	2,2	11	true	yes	no	no	Coil embolization aneurysm + carrier vessel	1
24	f	Overweight PAD Diabetes	iPDA	2,2	13	true	yes	no	no	Coil embolization aneurysm + carrier vessel	-
25	m	Overweight Hypertension Dyslipidemia	iPDA	4,2	38	true	yes	no	no	Coil embolization aneurysm + carrier vessel	2
26	m	Overweight Hypertension Dyslipidimia	GDA	3.9	31	true	no	no	yes	Coil embolization aneurysm + carrier vessel	-
27	f	Smoking Hypertenision PAD	sPDA	3.3	10	true	yes	no	no	Coil embolization aneurysm + carrier vessel	-
28	m	Overweight Hypertension Diabetes	iPDA	6	12	true	yes	no	no	Coil embolization aneurysm + carrier vessel	3
29	f	Smoking Overweight Hypertension Dyslipidemia	sPDA	2.8	11	true	yes	no	no	Coil embolization aneurysm + carrier vessel	1
30	f	Overweight Hypertension Dyslipidemia Diabetes	iPDA	2	6	pseudo	yes	yes	yes	Stenting CA + Coil embolization aneurysm + carrier vessel	-
31	f	Smoking Overweight Hypertension Dyslipidemia	iPDA	6.5	13	true	yes	no	yes	Stenting CA + Coil embolization aneurysm + carrier vessel	-

*AVR* aneurysm-to-vessel ratio, *CA* celiac artery, *GDA* gastroduodenal artery, *arcPDA* pancreaticoduodenal arcade, *iPDA* inferior pancreaticoduodenal artery, *sPDA* superior pancreaticoduodenal artery, *PAD* peripheral arterial disease

### Therapy assessment

Therapeutic planning was guided by the presence of CA stenosis > 50% or total occlusion, the position of the primary aneurysm (critical vs. non-critical), AVR, the presence of active bleeding or aneurysm rupture, and aneurysm type (e.g., pseudoaneurysm).

**Fig. 1:**
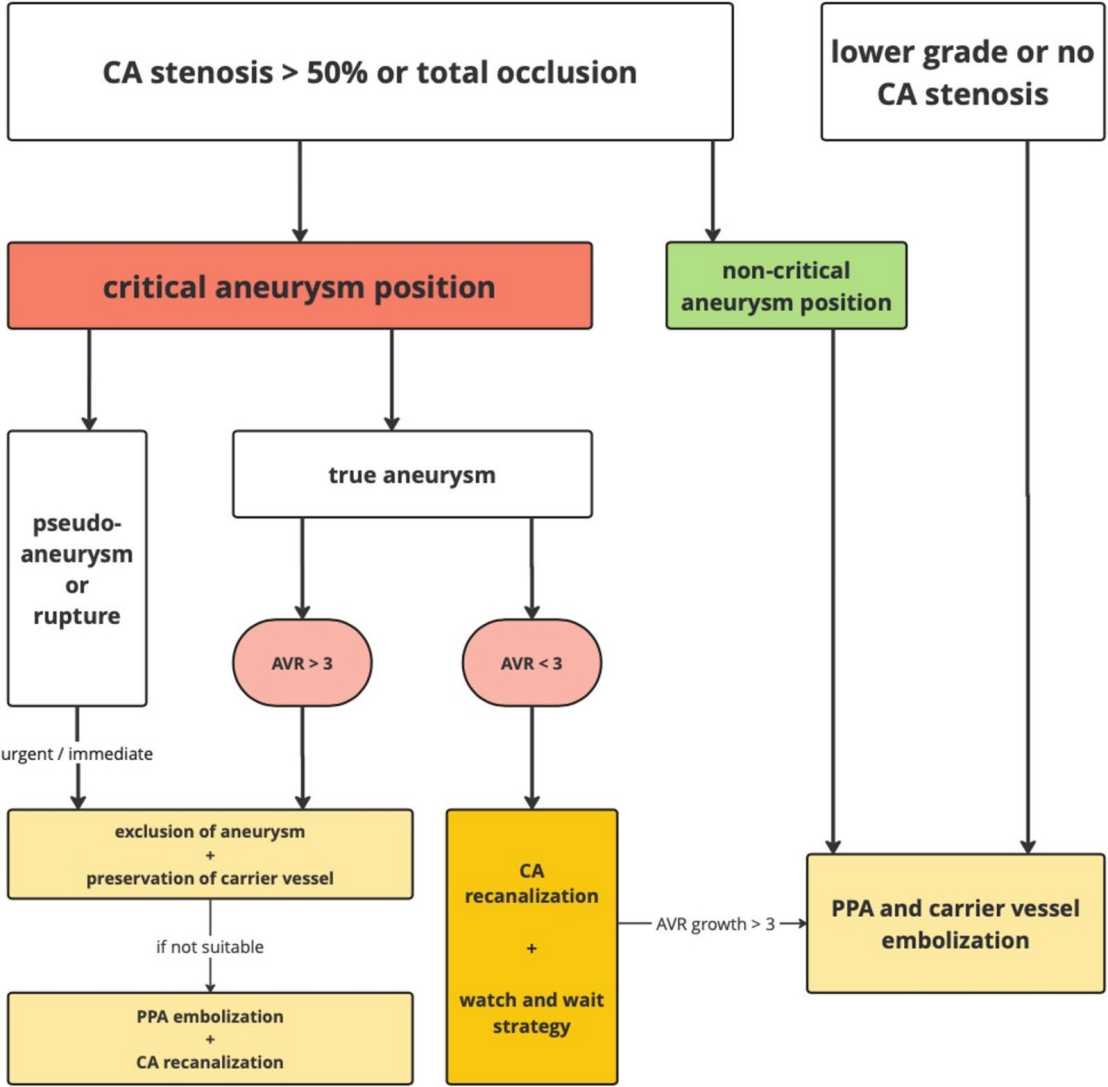
Therapy algorithm for PPA in case of CA stenosis. AVR = aneurysm-to-vessel ratio, CA = celiac artery, PPA = peripancreatic aneurysm

An overview of the therapeutic algorithm applied in this study is visualized in Fig. 1.

Out of the 31 patients, 5 (16.1%) had either no CA stenosis (4 patients) or low-grade stenosis (1 patient). These patients were treated with coil embolization in front-and-back-door technique of the primary aneurysm and the carrier vessel, regardless of the AVR or aneurysm location.

**Fig. 2:**
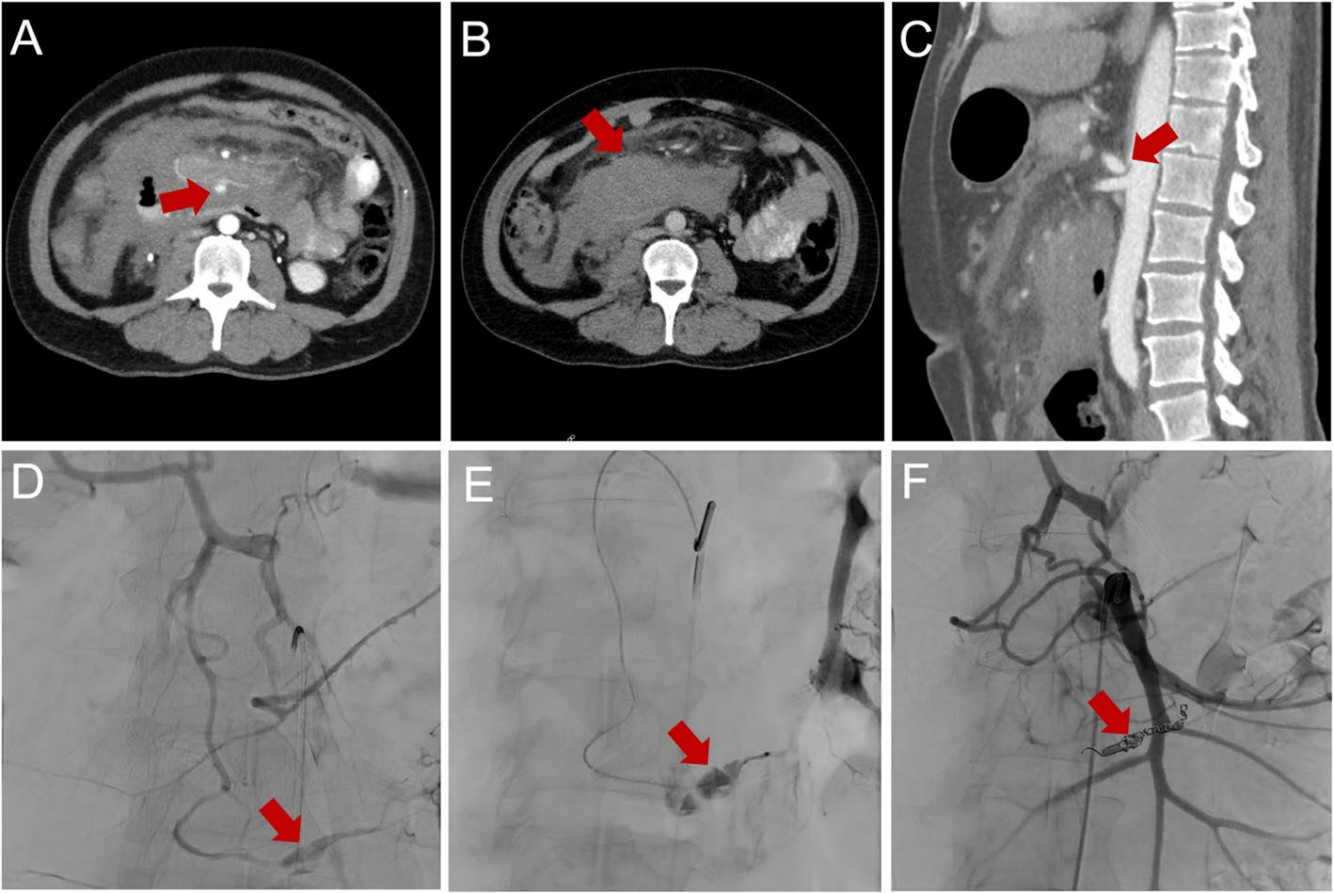
Representative case. MIP CTA and DSA imaging of a 44-year-old male patient with admission to our clinic with upper abdominal pain. **A** CTA showed high grade CA stenosis and a ruptured 10 mm pseudoaneurysm of the pancreaticoduodenal arcade in a non-critical position (red arrow) with (**B**) surrounding hematoma (red arrow). **D** Since probing the CA was extremely difficult at the beginning of the intervention, the initial findings were presented via mesentericography, where the well-known aneurysm was detected as a subtle contrast blush (red arrow). **E** Successful probing of the CA with a reversed curve catheter and super selective probing of the pancreaticoduodenal arcade. **F** Front-and-back-door coil-embolization of the pancreaticoduodenal arcade aneurysm was successful

In the remaining 26 patients, higher-grade CA stenosis or total occlusion was present. Among these, the primary aneurysm was non-critical in 16 patients (51.6%). These patients underwent front-and-back-door coil embolization of both the aneurysm and the carrier vessel, including 3 emergency cases with active bleeding. One patient with a non-critical aneurysm did not receive treatment due to a concurrent malignant tumor and was managed with palliative care. A representative case of a non-critical aneurysm is depicted in Fig. 2.

**Fig. 3:**
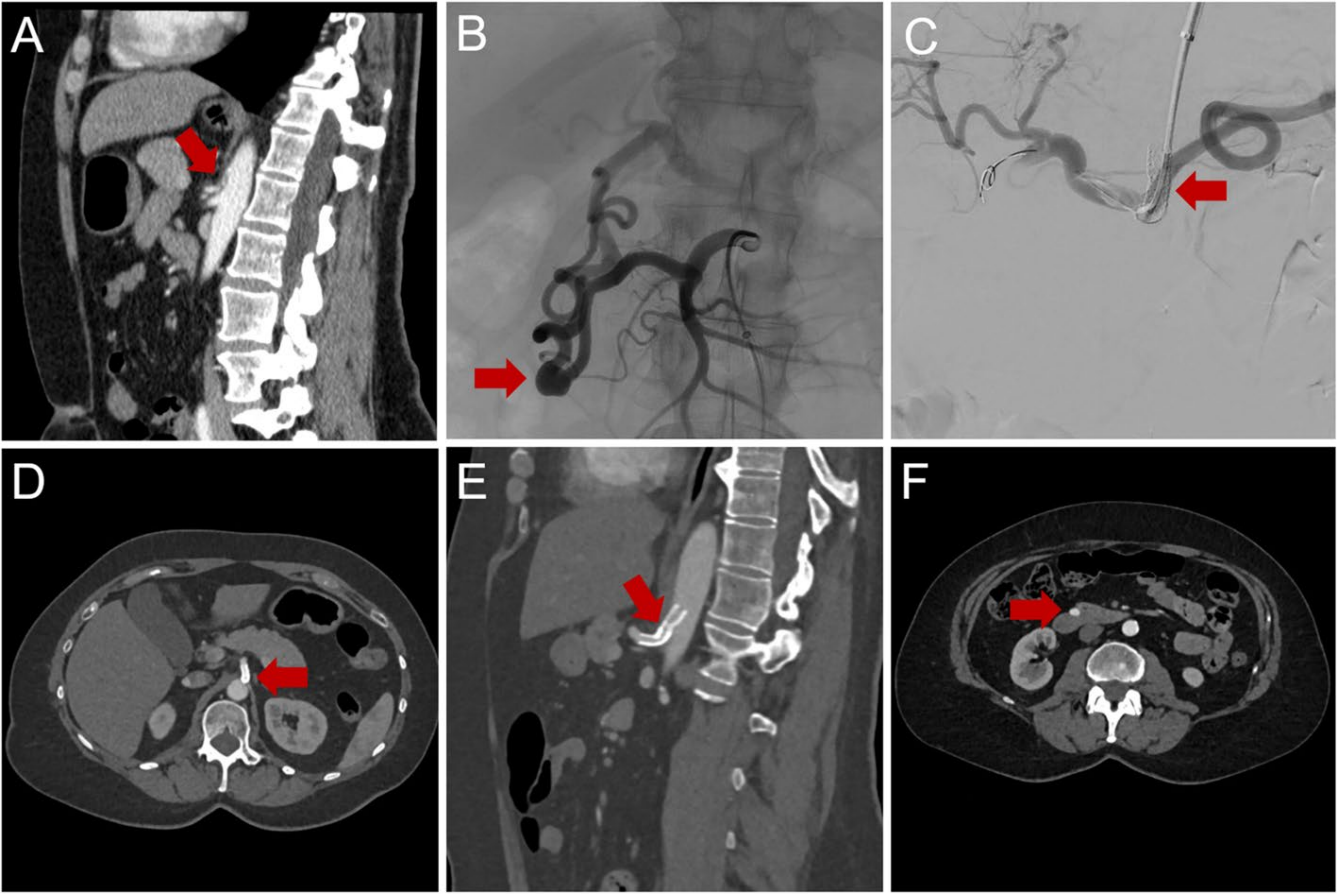
Representative case. CTA and DSA imaging of a 58-year-old female patient, who was referred to our clinic with upper abdominal pain. **A** Sagittal CTA reconstructions depict a subtotal occlusion of the CA (red arrow). **B** DSA showed a GDA aneurysm with an AVR of 2.6 (absolute size: 8 mm × 8 mm × 6 mm) in a critical position (red arrow). **C** Aneurysm rupture or active bleeding was not present. CA recanalization (red arrow) and a watch-and-wait strategy were performed. Follow-up CT was performed every 6 months in the first year and then every 12 months. **D**, **E**, **F** Follow-up CTA after 48 months shows patency of the CA stent (red arrows) and size constancy of the GDA aneurysm (absolute size: 8 mm × 8 mm × 6 mm) (red arrow)

In the remaining 10 patients, the primary aneurysm was located in a critical position. Among these, 3 patients presented with actively bleeding aneurysms. The treatment strategies varied: One patient received stent graft implantation. The second patient underwent front-and-back-door coil embolization of the aneurysm and carrier vessel, along with CA stenting. The third patient required surgical intervention, including aneurysm ligation, aortotruncal bypass, and MAL division, due to the infeasibility of an endovascular approach. Figure 3 shows a representative case of a pseudoaneurysm in a critical position.

The remaining 3 patients with critical aneurysms, an AVR > 3 and no signs of hemorrhage received aneurysm exclusion with preservation of the carrier vessel, using stent/coil and balloon remodeling techniques, and aneurysm embolization with stenting of the CA in one case.

**Fig. 4:**
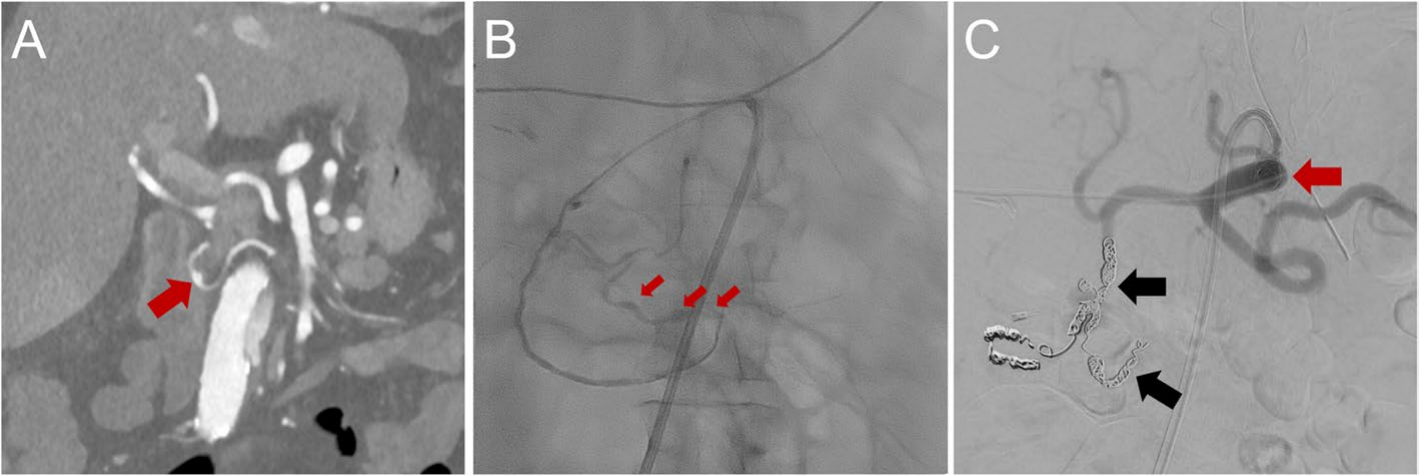
Representative case. CTA and DSA imaging of a 78-year-old female patient, who was referred to our clinic with recurrent duodenal bleeding. **A** CTA showed a CA stenosis > 50% and a 6 mm × 5 mm × 5 mm superior PDA pseudoaneurysm (red arrow) with an AVR of 2 in a critical position, there was no sign of an active bleeding. **B** DSA on the following day revealed an active contrast extravasation (red arrows) in position of the diagnosed aneurysm. **C** Coil embolization (black arrows) of the superior PDA aneurysm in front-and-back-door technique and CA stenting (red arrow) were performed

The remaining 4 patients with aneurysms in critical positions and AVR < 3 underwent CA revascularization followed by a watch-and-wait strategy for the aneurysm; endovascular stent implantation was performed in all four patients, with one also undergoing additional surgical MAL division. Follow-up CT was performed at 6, 12, 24 and 48 months after CA revascularization and showed no further growth or rupture of the PPA. Figure 4 depicts a representative case of a patient with an aneurysm in a critical position with an AVR < 3.

Secondary aneurysms did not meet the criteria of therapy.

## Discussion

In this retrospective observational study, the relationship between PPAs and VA stenosis was investigated, leading to the development of a treatment algorithm. It was conceptualized with careful consideration of aneurysm location, configuration, type, and the implications these factors have on therapeutic options. The main findings of this study demonstrate a high incidence of CA steno-occlusive lesions in patients with PPAs (87.1%) and a high correlation between the AVR and the incidence of aneurysm rupture.

The management of PPAs in patients with CA stenosis presents an ongoing clinical dilemma. Current Standards of Practice recommend treating PPAs of any size due to possible life-threatening complications [6, 20, 21]. Currently there are 226 published cases of PPAs in the literature [22]. Tailored treatment strategies considering aneurysm location, type and presence of accompanying VA stenosis are not available. In our study, we observed a high incidence of CA stenosis in 87.1% of patients with PPAs. This contrasts with prior studies, where the incidence of stenotic-occlusive VA lesions in those patients ranged between 55.8% and 75% [23, 24]. However, these earlier studies were limited by their small sample sizes [10, 11, 25, 26]. Despite the high prevalence of CA stenosis, current guideline-based recommendations do not adequately address the assessment of CA stenosis or the potential impact and complexities it introduces in PPA management [20, 21].The presence of VA stenosis complicates PPA management [6, 11], altered hemodynamics and increased collateral circulation may contribute to aneurysm formation and influence the risk of rupture [6, 20]. The altered blood flow dynamics may necessitate a different therapeutic approach compared to patients without such stenosis.

Recently, several studies have shown that the AVR provided a better prediction of rupture for smaller abdominal aortic aneurysms and intracranial aneurysms than the absolute aneurysm size [27–30]. The current data suggest that this relationship may also apply to PPAs. Specifically, our findings demonstrate that absolute aneurysm size does not correlate with the risk of rupture, whereas a higher AVR was associated with an increased risk of rupture. These insights highlight the importance of AVR as a relevant metric in assessing rupture risk in PPAs.

Further studies suggest that in abdominal aneurysms, the proportion of intraluminal thrombus offers a more accurate prediction of rupture risk than absolute size alone [31, 32]. Were unable to accurately calculate the ratios between the thrombus and aneurysm size due to the relatively small size of the PPAs.

When comparing the therapeutic regimens administered to patients in this study with the recommendations outlined in current guidelines, it becomes apparent that a significant proportion of patients were treated in ways that deviate from established guidelines. If the current recommendations [20, 21] had been strictly followed, all patients in this cohort would have received PPA embolization, a course of action that would likely have led to visceral ischemia. However, the unique constellation of this cohort, with over 80% of patients presenting with CA stenosis, necessitated alternative treatment strategies to prevent ischemic complications.

To address the issues not covered in current recommendations on PPA management, we developed a tailored algorithm for PPA treatment. This algorithm is based on the various treatment strategies performed on the current PPA collective, taking into account the unique clinical presentations and challenges observed in these cases.

The initial step in the algorithm is to determine whether the aneurysm is located in a critical or non-critical position.

Percutaneous embolization is the therapy of choice for PPAs situated in non-critical positions.

For PPAs in critical positions, therapy considerations become more complex, requiring the evaluation of three distinct scenarios:

- In the life-threatening situation of an actively bleeding aneurysm, immediate intervention is essential. The initial goal is to embolize the aneurysm while preserving the feeding vessel whenever possible. Due to the urgency, this approach is often not feasible. In such cases, primary embolization of the PPA is the preferred approach, followed by secondary recanalization of any associated VA stenosis. Pseudoaneurysms, regardless of their size and position, also require urgent treatment.
- The second scenario involves true aneurysms exceeding an AVR greater than 3, indicating a substantially increased risk of rupture. Accordingly, the recommended treatment approach parallels that of pseudoaneurysms. Unlike emergent cases, these interventions can be scheduled electively, allowing for thorough preprocedural planning and patient optimization [21]. Prior to addressing the aneurysm, recanalization of the VA stenosis should be performed to mitigate the risk of transient visceral ischemia during the procedure.
- The third scenario involves true aneurysms with an AVR < 3. In this case, a watch-and-wait strategy can be considered with regard to the PPA, while VA revascularization should be considered to potentially mitigate aneurysmal growth. The revascularization can be performed via an endovascular or open approach, depending on the underlying lesion. Monitoring of the PPA allows for differentiation of growing and stable subtypes. A CT check should be done every 6 months within the first year and every 12 months thereafter. Growing subtypes should then be treated, when the AVR exceeds 3.

This algorithm introduces a more differentiated approach for PPAs with accompanying CA stenosis.

While visceral arterial stenting is a viable option for revascularization, it is important to recognize that the patency rates of stents are not optimal. This is a critical consideration, especially when dealing with aneurysms in critical positions [21, 33]. In the event of stent occlusion, there is a significant risk of visceral ischemia. Recent data suggest that stent grafts offer improved patency rates [34, 35]. Therefore, an alternative treatment option is placement of stent grafts for VA stenosis followed by PPA embolization.

Since the incidence of SMA stenosis was low in our study and the SMA stenoses that occurred were low-grade, these were not included in the therapy algorithm.

### Limitations

Our study has limitations that must be acknowledged. As a retrospective observational study, it is inherently subject to biases associated with the reliance on historical data. Secondly, the small collective limits the generalizability of our findings, making it difficult to draw broad conclusions applicable to larger populations. Also, the statistical analysis is limited by the small collective. Additionally, the absence of follow-up data in a large portion of patients prevents us from assessing long-term outcomes and potential changes over time.

## Conclusions

Our study highlights the relationship between PPAs and VA stenoses, particularly in the CA. We found a high incidence of CA stenosis in PPA patients and showed that aneurysm rupture risk is more strongly linked to the AVR than size. This emphasizes the need for a more nuanced treatment approach, as current guidelines do not fully address the interaction between PPA features and arterial stenosis. We propose a differentiated treatment algorithm based on aneurysm location, rupture risk, and stenosis severity, offering a more personalized management strategy. While our findings support tailored interventions, especially for critical aneurysms, further large-scale studies are needed to validate and refine these approaches, ultimately improving patient outcomes.

## Data Availability

Not applicable.

## Abbreviations

AVR: Aneurysm-to-vessel ratio
CA: Celiac artery
GDA: Gastroduodenal artery
MAL: Median arcuate ligament
PDA: Pancreaticoduodenal arteries
PPA: Peripancreatic aneurysm
SMA: Superior mesenteric artery
VA: Visceral artery
